# Editorial

**Published:** 1904-06

**Authors:** 


### THE TEXAS MEDICAL JOURNAL.

AUSTIN, TEXAS.

A MONTHLY JOURNAL OF MEDICINE AND SURGERY.

EDITED AND PUBLISHED BY
F. E. DANIEL, M. D.
Mrs. F. E. DANIEL, Managing Editor.

ASSOCIATE EDITOR:
WITTEN BOOTH RUSS, M. D., San Antonio, Texas.

Published Monthly at Austin, Texas. Subscription price $1.00 a year in advance.
Eastern Representative: John Guy Monihan, St. Paul Building, 220 Broadway, New York City.
Official organ of the West Texas Medical Association, the Houston District Medical Association, the Austin District Medical Society, the Brazos Valley Medical Association, the Galveston County Medical Society, and several others.

Here Ends the Nineteenth Year of the phenomenal career of "The Famous Red Back." A success from the first, it is now recognized as a power in the cause of legitimate medicine and a pure and exalted profession. Since the reorganization, scores of new subscribers have "jined the army," and still they come. Every member should make it a point to not miss a single copy of volume 20, beginning July, 1904. Selah!

---

Have Manufacturing Pharmacists Any Rights that Should be Respected? Permit me to smile, please. Ask Siegel Cooper Company, the big department store fellows of New York what they know of pirating on Pepto-Mangan Gude, and if they do not now wish they hadn't? They have been selling a stuff they call "Pepto Manganate," put up in terra cotta wrappers, in imitation of the well known packages of M. J. Breitenbach Company, the manufacturers of Pepto-Mangan Gude. Breitenbach Company didn't do a thing to them. Oh, no; they knocked 'em out worse than the Japs did that other big robber at Kinchon. This is not the first time that Breitenbach Company have laid out burglars of the Seigel Cooper kind. About a year ago they put the fixin's on a fellow—I've forgotten his name. Here is what the company did for Seigel Cooper Company:

Ordered, adjudged and decreed as follows:

First. That the plaintiff, M. J. Breitenbach Company, is the owner of the sole and exclusive right to the use of the words "Pepto-Mangan" as a trade mark and trade name, as applied to medical preparations, throughout the United States and Canada, and has the sole and exclusive right in the same countries, of putting up and selling the preparation known as Gude's "Pepto-Mangan," according to the secret process and formula discovered by Dr. A. Gude, of Leipsiz.

Second. That the said defendants, the Siegel, Cooper Company and Thomas H. McInnerney, their agents, servants, employes and attorneys, be and they hereby are forever enjoined and restrained from making use of the words "Pepto-Manganate" in any manner whatsoever, either alone or in combination with other words, or from using the words "Pepto-Mangan," or any word or words similar to the words "Pepto-Mangan" in sound or appearance, in connection with the advertisement or sale or otherwise, of any medical or other preparation—excepting only that of the plaintiff.

Third. That the said defendants the Siegel, Cooper Company and Thomas H. McInnerney forthwith deliver to the plaintiff, or his attorney, to be destroyed, all bottles, packages, wrappers, circulars, or other things in their possession or under their control, or that of either of them, bearing the words "Pepto-Manganate," or any similar words.

Fourth. That the said plaintiff, The M. J. Breitenbach Company, recover of the said defendants, the Siegel, Cooper Company and Thomas H. McInnerney the damages, to be assessed by the court, resulting from the use by said defendants of the name "Pepto-Manganate," which is hereby adjudged to be a violation of the plaintiff's rights in the name "Pepto-Mangan."

Fifth. That the plaintiff recover of the said defendants the costs of this action.

Enter,

HENRY BISCHOFF,

Justice of the Supreme Court of the State of New York.

THOS. L. HAMILTON, Clerk.

No. 136. State of New York, County of New York.

I will add that it's a pity that the law does not make such stealing, for it is stealing, pure and simple, a penitentiary offense. It is about time that this cuckoo business was put a stop to. There is lots of substitution of this and other kinds done in Texas, notably in Houston, and the self-respecting doctors should boycott every substitutor. The substitutor holds in his dishonest hands the lives of the patients, and the honor and career of the prescribing physician. He can kill the first and wreck the latter. I had a bill introduced in the last Legislature to make substituting on a doctor's prescription a penal offense—fine and imprisonment. There came a flood of petitions against it from so-called respectable retail druggists all over Texas—principally from Houston. They came in such quantity that the Speaker of the House would never let Dr. Miller (representative from Milam) bring up the bill. Please the Lord, I'll try it again next session.

Dr. Pollock, representing the New York Pharmacal Company, caught a leading druggist in Houston in the act of putting up "something just as good" for Viburnum Compound, Hayden.

ANOTHER KNOCK-OUT comes to me from London. The English courts, too, put the fixin's on the imitator and substitutor, and upheld the rights of the manufacturing pharmacist to protection under the trade-mark laws. Burroughs, Welcome & Co., who invented and patented the word "Tabloid"—their trade mark—brought suit against Thompson & Capper for "passing off goods other than those of the plaintiffs', when the trade mark 'Tabloid' was used on prescriptions or orders." This case was on trial a week. Over 500 witnesses were summoned from all over Europe. It attracted much attention, and the decision has been favorably commented upon by the press of London, Berlin, Vienna, Paris and New York.

The court ordered perpetual injunction against defendants, and that they pay damages and all costs.

Fairchilds Bros. & Foster, of New York, are sole American agents for Burroughs, Welcome & Co.'s Tabloid preparations.

STILL ANOTHER.—Fairchilds Bros. & Foster, of New York, the well-known pharmaceutical manufacturers, recently obtained a perpetual injunction against Morris Dlugasch and Herman Finkelstein (The Broadway Drug Company) from selling, dispensing, advertising or displaying at the drug store of said defendants, at No. 177 Broadway, Borough of Manhattan, City of New York, or elsewhere, any chemical or pharmaceutical preparations of any sort or kind whatsoever bearing signs, labels or wrappers marked "Fairchild" or "Dr. Fairchild," or any similar word or words, or purporting to be made by "Dr. Fairchild" or "Fairchild," which said preparations are not manufactured by plaintiff.

Is a doctor "in good standing" who repudiates and refuses to pay a small subscription bill, or any other honest debt? It is almost incredible, but it is neverthless true, that there are amongst the members of the State Medical Association—members of county medical societies—men, some of whom are prominent and well known and well to do physicians, who have taken the "Red Back" year after year, who totally ignore my little polite reminders and utterly defeat every effort at collection, even through a Chicago collecting agency. Some of them owe me from $5.00 to $15, and they are deaf and dead to every kind of appeal. Some of these fellows were here at the big meeting, some delegates, perhaps, or holding some office. I am awfully tempted to give some of their names. Sometimes an insurance company asks me about the Texas doctors who apply for the appointment of examiner. One of the usual questions is: "Does he pay his debts?" Of course, I have long since ceased sending the Journal to all who were in arrears five years; but, there are many to whom I still send it who owe two to four years, and to whom bills are sent four times a year, without eliciting any reply. It is an awful bore and labor and expense to make out and mail and pay postage on these bills every three months. In many instances, it is neglect; but it is not excusable; but, in many other instances, it is pure cussedness and deadbeatism. Every fellow who reads this will know if the cap fits him. The great mass of my readers are, however, as true as steel, and ante, regularly, every year. May they live long and prosper. "Please remit." "Be good." "Do it now."

